# Pregex: Rule-Based Detection and Extraction of Twitter Data in Pregnancy

**DOI:** 10.2196/40569

**Published:** 2023-02-09

**Authors:** Ari Z Klein, Shriya Kunatharaju, Karen O'Connor, Graciela Gonzalez-Hernandez

**Affiliations:** 1 Department of Biostatistics, Epidemiology, and Informatics Perelman School of Medicine University of Pennsylvania Philadelphia, PA United States; 2 Department of Computational Biomedicine Cedars-Sinai Medical Center West Hollywood, CA United States

**Keywords:** natural language processing, data mining, social media, pregnancy

## Introduction

Data on potential risk factors in pregnancy are limited. Meanwhile, in the United States, 17% of pregnancies end in fetal loss [1], and birth defects and preterm births are the leading causes of infant mortality [2]. In previous work [3], we developed an automated natural language processing pipeline that identifies users who announced their pregnancy on Twitter and collects all of their tweets on an ongoing basis. We have also demonstrated that their tweets can be used for observational studies [4-6]. However, selecting users for such studies involves additional processing to address a limitation of our pipeline—namely, that many of the users refer to a pregnancy either that occurred prior to the availability of their tweets or for which we could not determine the prenatal period. To streamline the use of Twitter as a source of data, the objective of this study was to advance a downstream system developed in our previous work [7] and evaluate its upstream use for identifying tweets that indicate the availability of Twitter data during pregnancy and can be used to extract dates marking the beginning and end of the 40-week prenatal period.

## Methods

### Ethical Considerations

The data used in this study were collected in accordance with the Twitter Terms of Service. The institutional review board of the University of Pennsylvania reviewed this study and deemed it exempt human subjects research under 45 CFR §46.101(b)(4) for publicly available data sources.

### Natural Language Processing System

Our system, Pregex [8], uses more than 100 handwritten regular expressions to search for tweets in which users indicate their gestational age or due date, including as units of time, days of the week, numeric and spelled-out dates, and linguistic markers. We took an iterative approach [9] to develop the regular expressions, allowing us to actively reduce noise and account for ways that this information may be presented on Twitter, including in hashtags and with lexical variants [10]. Table 1 presents sample matching tweets.

Pregex uses the *dateutil* Python package to apply an arithmetic operation to the tweets’ timestamp, based on the regular expression that the tweets match. For tweet 1, after replacing *5 1/2 mos* with *5 months and 2 weeks* in preprocessing, Pregex assigns the first digit group (*5*) to the *months* parameter of the *relativedelta* function and the second digit group (*2*) to the *weeks* parameter, subtracts this *relativedelta* from the timestamp to calculate the start date of the 40-week prenatal period, and then adds *40 weeks* to the start date to calculate the due date. For tweet 2, Pregex assigns *Saturday* to the *weekday* parameter to calculate the due date and then subtracts *40 weeks* from the due date to calculate the start date.

**Table 1:** Sample tweets detected by Pregex (matching pattern in italics).

Tweet	Timestamp	Pregnancy start	Pregnancy end
*I am 5 1/2 mos pregnant* & severely anemic. I had hypermesis in my first trimester.	November 11, 2020	June 9, 2020	March 16, 2021
*My due date is Saturday* and I hope my baby boy is ready to come on out lol	January 23, 2020	April 20, 2019	January 25, 2020
Is October too early to have a baby shower when *I’m due Feb 8th*? I want a Halloween themed baby shower	July 23, 2020	May 4, 2020	February 8, 2021
I can’t wait until my pregnancy pillow comes, having the worst nights sleep *#21weekspregnant*	April 18, 2020	November 23, 2019	August 29, 2020
i can’t believe *i’m already half way through my pregnancy*, this heat is really starting to get to me now	June 19, 2021	January 30, 2021	November 6, 2021

## Results

We deployed Pregex on the Twitter timelines of more than 550,000 users—mostly users identified by our original pipeline [3]—and detected approximately 235,000 tweets that were posted by more than 100,000 users. For validation, 3 annotators labeled a random sample of 4017 matching tweets—1 tweet per user and up to 100 tweets per regular expression—to identify whether they self-report an ongoing pregnancy, and the correct beginning and end dates were extracted (Multimedia Appendix 1). Among these 4017 tweets, 3716 (90%) were dual annotated and 400 (10%) were annotated by all 3 annotators. For 381 (95%) of these 400 tweets, the 3 annotators agreed on whether the tweet self-reports an ongoing pregnancy, agreeing that 378 (99%) of these 381 tweets do. For 376 (99%) of these 378 tweets, the 3 annotators agreed on whether the correct beginning and end dates were extracted. After resolving disagreements among all 4017 tweets, we established that Pregex had a precision of 0.96 for identifying ongoing pregnancies, where *precision = true positives / (true positives + false positives)*. Among the 3875 true positives, Pregex had a precision of 0.99 for extracting dates marking the beginning and end of the 40-week prenatal period.

## Discussion

Because pregnancy is a common event, our rule-based approach can identify tweets during pregnancy with high precision and on a large scale, facilitating the use of Twitter as a complementary source of data for observational studies. In real time, Pregex is detecting approximately 50 new users daily, taking as input approximately 15,000 tweets returned from the Twitter streaming application programming interface that matches pregnancy-related keywords derived from the regular expressions (Multimedia Appendix 2). Among the 142 false-positive tweets in our evaluation that did not self-report an ongoing pregnancy, 42 (29%) mention a due date that refers to a deadline (eg, payments), which we will address in future work.

## Appendix

Multimedia Appendix 1 Annotated tweets for evaluation.

Multimedia Appendix 2 Pregnancy-related keywords for Twitter streaming application programming interface.
